# Symmetrical Root Enlargement Following Straight Longitudinal Aortotomy via a Right Anterior Minithoracotomy

**DOI:** 10.1093/icvts/ivaf275

**Published:** 2025-11-21

**Authors:** Naonori Kawamoto, Kizuku Yamashita, Kota Suzuki, Satsuki Fukushima

**Affiliations:** Cardiac Surgery, National Cerebral and Cardiovascular Center, Suita 564-8565, Japan; Cardiac Surgery, National Cerebral and Cardiovascular Center, Suita 564-8565, Japan; Cardiac Surgery, National Cerebral and Cardiovascular Center, Suita 564-8565, Japan; Cardiac Surgery, National Cerebral and Cardiovascular Center, Suita 564-8565, Japan

**Keywords:** straight longitudinal aortotomy, right anterior minithoracotomy, symmetrical root enlargement

## Abstract

Root enlargement via traditional transverse or oblique aortotomy disrupts the anatomical features of smooth continuity and symmetry from the aortic root to the proximal ascending aorta. A straight longitudinal aortotomy, extended vertically into nadir of noncoronary aortic annulus, achieves smooth continuity and symmetrical enlargement from the aortic root to the proximal ascending aorta with a tear-drop-shaped bovine pericardial patch. Herein, we report successful symmetrical root enlargement following straight longitudinal aortotomy via right anterior minithoracotomy.

## INTRODUCTION

We previously reported that straight longitudinal aortotomy has advantages regarding suturing and hemostasis for right anterior minithoracotomy.^1^

Extension of a straight longitudinal aortotomy bisects the noncoronary sinus. Subsequent root enlargement leads to symmetrical expansion of the aortic root, sinotubular junction (STJ), and proximal ascending aorta. Herein, we describe a case of symmetrical root enlargement following straight longitudinal aortotomy via a right anterior minithoracotomy.

**Figure 1.**
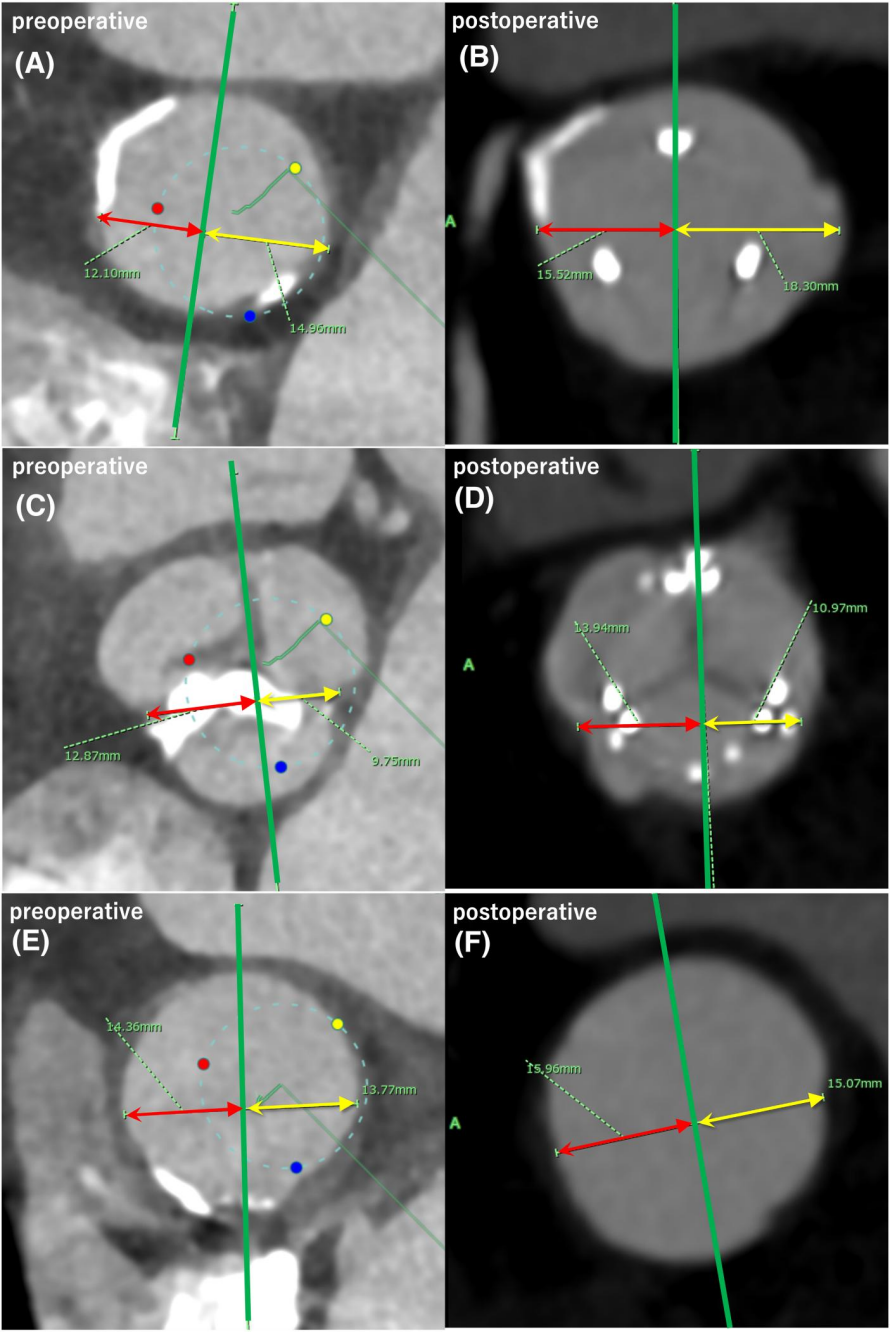
Cardiac Measurements on Computed Tomography Angiography Images. Each Image Pair Indicates Pre- and Postoperative Images, Respectively. (A and B) Sinotubular Junction Level; the Red (Left from Center) and Yellow (Right from Center) Lines Indicate Increases of 3.42 and 3.34 mm, Respectively. (C and D) Valsalva Sinus Level; the Red and Yellow Lines Indicate Increases of 1.07 and 1.22 mm, Respectively. (E and F) Proximal Ascending Aortic Level; the Red and Yellow Lines Indicate Increases of 1.6 and 1.3 mm, Respectively. Green Line: Center Line from the Commissure Between The Left and Right Coronary Sinuses to the Nadir of the Noncoronary Cusp

A 75-year-old woman with severe aortic stenosis had a mean gradient of 62 mmHg, 19-mm annulus, 24-mm STJ, and 27-mm Valsalva sinus. After root enlargement following straight longitudinal aortotomy, a 21-mm Inspiris (Edwards Lifesciences LLC) aortic bioprosthesis was implanted. Postoperative computed tomography angiography showed the root, STJ, and proximal ascending aorta adequately enlarged symmetrically compared with the preoperative images (**Figure 1**).

## ROOT ENLARGEMENT

**Figure 2.**
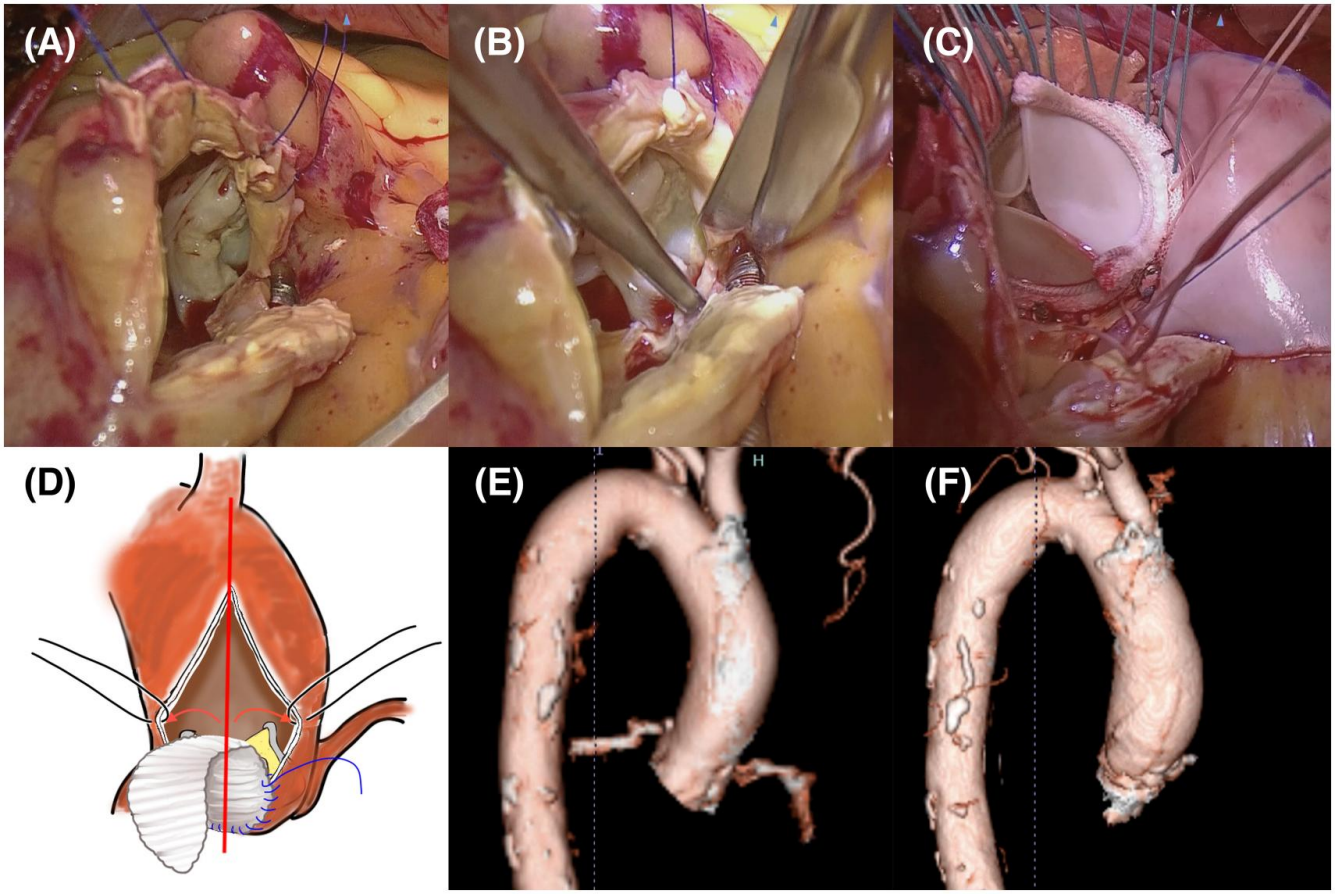
Symmetrical Root Enlargement Technique following Straight Longitudinal Aortotomy via a Right Anterior Minithoracotomy (A) A Straight Longitudinal Incision Was Made in the Lateral Aspect of the Ascending Aorta. (B) The Longitudinal Incision Was Continued into the NCC Annulus. (C) Noneverting Sutures Were Placed along the Native Aortic Annulus Including Three Sutures Placed from Outside-in on the Bovine Patch. (D) Schematic of the Straight Longitudinal Incision From the Ascending Aorta into the Annulus at the Nadir of the Noncoronary Cusp. (E and F) Preorerative and Postoperative Three-Dimensional Reconstruction Images Showing That Root Enlargement following Straight Longitudinal Aortotomy Preserves Natural Continuity and Symmetry from the Aortic Root to the Proximal Aorta

After cardiac arrest, a straight longitudinal incision was made in the lateral aspect of the ascending aorta 3–5 cm below the origin of the brachiocephalic artery (**Figure 2A**), guided by preoperative computed tomography. The incision was extended into the nadir of the noncoronary sinus, bisecting the noncoronary sinus. The aortic valve was excised, the annulus was debrided, and the incision was continued into the noncoronary cusp (NCC) annulus without involving the mitral curtain (**Figure 2B**). A tear-drop-shaped bovine pericardial patch (Maquet; Getinge Group, Getinge AB, Göteborg, Sweden) was fashioned. One end of the patch was sewn with 5–0 Prolene (Ethicon Inc., Somerville, NJ, USA) running sutures to the distal end of the enlargement at the level of the aortic annulus. Both limbs of the suture were passed beyond the native STJ level and secured. The position of the sizer on the patch was marked to guide the placement of the valve sutures. Noneverting, pledgetted 2–0 ETHIBOND sutures (Ethicon Inc.) were placed along the native aortic annulus. Three sutures were placed from outside-in on the bovine patch. The bioprosthesis was placed with one strut facing the left-to-right commissural post (**Figure 2C**). The aortotomy was then fully closed. Operation and cardiac arrest times were 272 min and 99 min, respectively.

## DISCUSSION

Root enlargement following traditional transverse or oblique aortotomy disrupts the anatomical features of smooth continuity and symmetry from the aortic root to the proximal ascending aorta. Yang et al^2^ previously reported that partial aortotomy for Y-incision root enlargement did not always enlarge the proximal ascending aorta. Therefore, the roof technique for Y-incision root enlargement was developed to achieve smooth enlargement from the aortic root to the proximal ascending aorta. However, this approach requires multiple suture lines compared with traditional root enlargement. Additionally, suturing and hemostasis are complex, which makes this technique unsuitable for minimally invasive surgery. Matsumoto et al^3^ performed Y-incision and roof techniques for aortic root enlargement via right minithoracotomy and reported a mean cardiac arrest time of >3 hours.

We previously reported straight longitudinal aortotomy, which has advantages in right minithoracotomy.^1^ The incision line is minimal and near the skin incision; therefore, hemostasis and closing the aortotomy are simple and safe. The incision line, extended vertically into the NCC nadir, also incises the aortic root and proximal aorta, achieving smooth continuity and symmetrical enlargement of the aortic root with a tear drop-shaped bovine pericardial patch (**Figure 2D-F**). Our procedure essentially enlarges the basal portion of the aortic root rather than the annulus itself. Accordingly, in contrast to the Y-incision technique, it does not permit upsizing of the prosthetic valve by more than two sizes. Nevertheless, our procedure is technically straightforward and maintains geometric symmetry, and therefore may represent a potential alternative option in the root enlargement of patients with a small aortic root.

## CONCLUSION

Root enlargement following straight longitudinal aortotomy is safe and feasible for right anterior minithoracotomy aortic valve replacement. This approach achieves smooth continuity and symmetry from the aortic root to the proximal ascending aorta.

## Data Availability

The data used in this case report were obtained from the hospital’s internal platform and are not publicly available due to patient privacy and data protection regulations. Interested researchers may request access to the anonymized data through the hospital’s archival system, subject to ethical approval and review by the relevant institutional review board.
